# Supplementary material for: Patient-Specific Vascularized Tumor Model: Blocking TAM Recruitment with Multispecific Antibodies Targeting CCR2 and CSF-1R
Source: bioRxiv. 2023 Nov 29:2023.11.28.568627. Preprint. [Version 1] doi: 10.1101/2023.11.28.568627 (PMC10705378; doi:10.1101/2023.11.28.568627)
Supplement: 1 [file NIHPP2023.11.28.568627V1-supplement-1.pdf]

bioRxiv preprint doi: https://doi.org/10.1101/2023.11.28.568627; this version posted November 29, 2023. The copyright holder for this preprint (which was not certified by peer review) is the author/funder, who has granted bioRxiv a license to display the preprint in perpetuity. It is made available under aCC-BY 4.0 International license.

Table S1: Primer sequences for real-time RT-PCR

**Gene name**	**Sequences**	**Gene name**	**Sequences**
GAPDH Forward	GTCTCCTCTGACTTCAACAGCG	CCL8 Forward	TATCCAGAGGCTGGAGAGCTAC
GAPDH Reverse	ACCACCCTGTTGCTGTAGCCAA	CCL8 Reverse	TGGAATCCCTGACCCATCTCTC
TGF-β Forward	TACCTGAACCCGTGTTGCTCTC	CCL2 Forward	AGAATCACCAGCAGCAAGTGTCC
TGF-β Reverse	GTTGCTGAGGTATCGCCAGGAA	CCL2 Reverse	TCCTGAACCCACTTCTGCTTGG
CD-80 Forward	CTCTTGGTGCTGGCTGGTCTTT	CCL13 Forward	GATCTCCTTGCAGAGGCTGAAG
CD-80 Reverse	GCCAGTAGATGCGAGTTTGTGC	CCL13 Reverse	TCTGGACCCACTTCTCCTTTGG
CD-206 Forward	AGCCAACACCAGCTCCTCAAGA	CCL7 Forward	ACAGAAGGACCACCAGTAGCCA
CD-206 Reverse	CAAAACGCTCGCGCATTGTCCA	CCL7 Reverse	GGTGCTTCATAAAGTCCTGGACC
M-CSF Forward	TGAGACACCTCTCCAGTTGCTG	IL-1B Forward	CCACAGACCTTCCAGGAGAATG
M-CSF Reverse	GCAATCAGGCTTGGTCACCACA	IL-1B Reverse	GTGCAGTTCAGTGATCGTACAGG
Il-10 Forward	TCTCCGAGATGCCTTCAGCAGA		
Il-10 Reverse	TCAGACAAGGCTTGGCAACCCA		

Predesigned Primetime qPCR primer pairs for GAPDH (Hs.PT.39a.22214836), CSF-1R (Hs.PT.58.3041870), M-CSF (Hs.PT.58.26882150), IL34 (Hs.PT.58.39625359) were purchased from IDT.
